# A Multi-Strategy Adaptive Comprehensive Learning PSO Algorithm and Its Application

**DOI:** 10.3390/e24070890

**Published:** 2022-06-28

**Authors:** Ye’e Zhang, Xiaoxia Song

**Affiliations:** College of Computer and Network Engineering, Shanxi Datong University, Datong 037009, China; sxdt_zye@sxdtdx.edu.cn

**Keywords:** CLPSO, multi-strategy, search ability, photovoltaic optimization

## Abstract

In this paper, a multi-strategy adaptive comprehensive learning particle swarm optimization algorithm is proposed by introducing the comprehensive learning, multi-population parallel, and parameter adaptation. In the proposed algorithm, a multi-population parallel strategy is designed to improve population diversity and accelerate convergence. The population particle exchange and mutation are realized to ensure information sharing among the particles. Then, the global optimal value is added to velocity update to design a new velocity update strategy for improving the local search ability. The comprehensive learning strategy is employed to construct learning samples, so as to effectively promote the information exchange and avoid falling into local extrema. By linearly changing the learning factors, a new factor adjustment strategy is developed to enhance the global search ability, and a new adaptive inertia weight-adjustment strategy based on an S-shaped decreasing function is developed to balance the search ability. Finally, some benchmark functions and the parameter optimization of photovoltaics are selected. The proposed algorithm obtains the best performance on 6 out of 10 functions. The results show that the proposed algorithm has greatly improved diversity, solution accuracy, and search ability compared with some variants of particle swarm optimization and other algorithms. It provides a more effective parameter combination for the complex engineering problem of photovoltaics, so as to improve the energy conversion efficiency.

## 1. Introduction

Many problems in reality can be transformed into optimization problems. These optimization problems have complex characteristics, such as multiple constraints, high dimensionality, nonlinearity, and uncertainty, making them difficult to solve by the traditional optimization methods [1,2]. Therefore, an efficient new method is sought to solve these complex problems. Swarm intelligence optimization algorithms are a new evolutionary computing technology, which refers to some intelligent optimization algorithms with distributed intelligent behavior characteristics inspired by the swarm behavior of insects, herds, birds, fish, etc. [3,4,5]. This has become the research focus of more and more researchers. It has a special relationship with artificial life, and includes Harris hawk optimization (HHO), slime mold algorithm (SMA), artificial bee colony (ABC), firefly optimization, cuckoo search, and brainstorming optimization algorithm [6,7,8,9] for engineering scheduling, image processing, the traveling salesman problem, cluster analysis, and logistics location.

PSO is a swarm intelligence optimization technology developed by Kennedy and Eberhart [10]. The main idea is to solve the optimization problem through individual cooperation and information sharing. The PSO takes on a simple, strong parallel structure. Therefore, it has been used in multi-objective optimization, scheduling optimization, vehicle routing problems, etc. Although the PSO shows good optimization performance, it has slow convergence in solving complex optimization problems. Thus, a variety of improvement strategies for PSO are presented. Nickabadi et al. [11] presented a new adaptive inertia weight approach. Wang et al. [12] presented a self-adaptive learning model based on PSO for solving application problems. Zhan et al. [13] presented an orthogonal learning strategy for PSO. Li and Yao [14] presented a cooperative PSO. Xu [15] presented an adaptive tuning for the parameters of PSO based on a velocity and inertia weight strategy to avoid the velocity close to zero in the early stages. Wang et al. [16] presented a hybrid PSO using a diversity mechanism and neighborhood search. Chen et al. [17] presented an aging leader and challenger PSO. Qu et al. [18] presented a distance-based PSO. Cheng and Jin [19] presented a social learning PSO based on controlling dimension-dependent parameters. Tanweer et al. [20] presented a self-regulating PSO with the best human learning. Taherkhani et al. [21] presented an adaptive PSO approach. Moradi and Gholampour [22] presented a hybrid PSO based on a local search strategy. Gong et al. [23] developed a new hybridized PSO framework with another optimization method for “learning”. Nouiri et al. [24] presented an effective and distributed PSO. Wang et al. [25] presented a hybrid PSO with adaptive learning to guarantee exploitation. Aydilek [26] presented a hybrid PSO with a firefly algorithm mechanism. Xue et al. [27] presented a self-adaptive PSO. Song et al. [28] presented a variable-size cooperative co-evolutionary PSO with the idea of “divide and conquer”. Song et al. [29] presented a bare-bones PSO with mutual information.


**Sources**

**Results and Contribution to PSO**
Nickabadi et al. [11]Designed an adaptive inertia weight strategy for PSOZhan et al. [13]Designed an orthogonal learning strategy for PSOXu [15]Designed an adaptive tuning strategy for the parameters for PSOWang et al. [16]Developed a hybrid PSOCheng and Jin [19]Developed a social learning PSOTanweer et al. [20]Developed a self-regulating PSOMoradi and Gholampour [22]Designed a local search strategy for PSOGong et al. [23]Developed a new hybridized PSOXue et al. [27]Developed a self-adaptive PSOSong et al. [28]Developed a variable-size cooperative co-evolutionary PSOSong et al. [29]Developed a bare-bones PSO

The comprehensive learning PSO (CLPSO) algorithm is a variant of PSO, and has good application in multimodal problems. However, because the CLPSO algorithm uses the current search velocity and individual optimal value to update the search velocity, the search velocity value in the later iteration is very small, resulting in slow convergence and reducing the computational efficiency. In order to improve the CLPSO algorithm, researchers have conducted some useful works. Liang et al. [30] presented a variant of PSO (CLPSO) using a new learning strategy. Maltra et al. [31] presented a hybrid cooperative CLPSO by cloning fitter particles. Mahadevan and Kannan [32] presented a learning strategy for PSO to develop a CLPSO to overcome premature convergence. Ali and Khan [33] presented an attributed multi-objective CLPSO for solving well-known benchmark problems. Hu et al. [34] presented a CLPSO-based memetic algorithm. Zhong et al. [35] presented a discrete CLPSO with an acceptance criterion of SA. Lin and Sun [36] presented a multi-leader CLPSO based on adaptive mutation. Zhang et al. [37] presented a local optima topology (LOT) structure with the CLPSO for solving various functions. Lin et al. [38] presented an adaptive mechanism to adjust the comprehensive learning probability of CLPSO. Wang and Liu [39] presented a novel saturated control method for a quadrotor to achieve three-dimensional spatial trajectory tracking with heterogeneous CLPSO. Cao et al. [40] presented a CLPSO with local search. Chen et al. [41] presented a grey-wolf-enhanced CLPSO based on the elite-based dominance scheme. Wang et al. [42] presented a heterogeneous CLPSO with a mutation operator and dynamic multi-swarm. Zhang et al. [43] presented a novel CLPSO using the Bayesian iteration method. Zhou et al. [44] presented an adaptive hierarchical update CLPSO based on the strategies of weighted synthesis. Tao et al. [45] presented an enhanced CLPSO with dynamic multi-swarm.


**Sources**

**Results and Contribution to PSO**
Maltra et al. [31]Developed a hybrid cooperative CLPSOAli and Khan [33]Developed an attributed multi-objective CLPSOHu et al. [34]Presented a CLPSO with local searchZhong et al. [35]Presented a discrete CLPSOLin and Sun [36]Designed an adaptive mutation for multi-leader CLPSOLin et al. [38]Designed an adaptive mechanism for CLPSOCao et al. [40]Developed a CLPSO with local searchChen et al. [41]Developed a grey-wolf-enhanced CLPSOWang et al. [42]Developed a heterogeneous CLPSOZhou et al. [44]Developed an adaptive hierarchical update CLPSOTao et al. [45]Developed an enhanced CLPSO with dynamic multi-swarm

These improved CLPSO algorithms use the individual optimal information of particles to guide the whole iterative process, have better diversity and search range, and can solve complex multimodal problems. However, because the global optimal value does not participate in the particle velocity and position, the particle velocity is too small in the later search, and the convergence speed is slow. At the same time, due to the lack of measures for avoiding the local optimization, once the optimal values of most particles fall into the local optimization, the convergence is unable to find the global optimal value, and the performance is unstable. Therefore, to improve the optimization performance of CLPSO, a novel multi-strategy adaptive CLPSO (MSACLPSO) based on making use of comprehensive learning, multi-population parallel, and parameter adaptation was designed for this paper. The MSACLPSO effectively promotes information exchange in different dimensions, ensures information sharing in the population, enhances the convergence and stability, and balances the search ability compared with the other related algorithms.

The main contributions and novelties of this paper are described as follows.

(1) A novel multi-strategy adaptive CLPSO (MSACLPSO) based on comprehensive learning, multi-population parallel, and parameter adaptation is presented.
(2) A multi-population parallel strategy is designed to improve population diversity and accelerate convergence.
(3) A new velocity update strategy is designed by adding the global optimal value in the population to the velocity update.
(4) A new adaptive adjustment strategy of learning factors is developed by linearly changing the learning factors.
(5) A parameter optimization method of photovoltaics is designed to prove the actual application ability.

## 2. PSO

PSO is a population-based search algorithm that simulates the social behavior of birds within a range. In PSO, all individuals are referred to as particles, which are flown through the search space to delete the success of other individuals. The position of particles changes according to the individual’s social and psychological tendencies. The change of one particle is influenced by knowledge or experience. As a modeling result of the social behavior, the search is processed to return to previously successful areas in the search space. The particle’s velocity (v) and position (x) are changed by the particle best value (pBest) and global best value (gBest). The formula for updating velocity and position is given as follows:

(1) $$v_{ij}^{t+1}=\omega v_{ij}^{t}+c_{1}r_{1}\left(pBest_{ij}^{t}-x_{ij}^{t}\right)+c_{2}r_{2}\left(gBest_{ij}^{t}-x_{ij}^{t}\right)$$

(2) $$x_{ij}^{t+1}=x_{ij}^{t}+v_{ij}^{t+1}$$

where $v_{ij}^{t+1}$ is the velocity of the $i^{th}$ particle at the $j^{th}$ iteration, $x_{ij}^{t+1}$ is the position of particle $i^{th}$ at the $j^{th}$ iteration, and the position of the particle is related to its velocity. w is an inertia weight factor, which is used to reflect the motion habits of particles and represent the tendency of particles to maintain their previous speed. $c_{1}$ is a self-cognition factor, which reflects the particle’s memory of its own historical experience, and represents that the particle has a tendency to approach its best position. $c_{2}$ is a social cognition factor, which reflects the population’s historical experience of collaboration and knowledge sharing among particles, and represents that particles tend to approach the best position in the population or field history. $r_{1}$ and $r_{2}$ represent random numbers in [0, 1], which denote the remembrance ability for the research. Generally, the value in the V can be clamped to the range [−$V_{\max}$, $V_{\max}$] in order to control the excessive roaming of particles outside the search space. The PSO is terminated until the maximal number of iterations is reached or the best particle position cannot be further improved. The PSO achieves better robustness and effectiveness in solving optimization problems.

**Figure 1:**
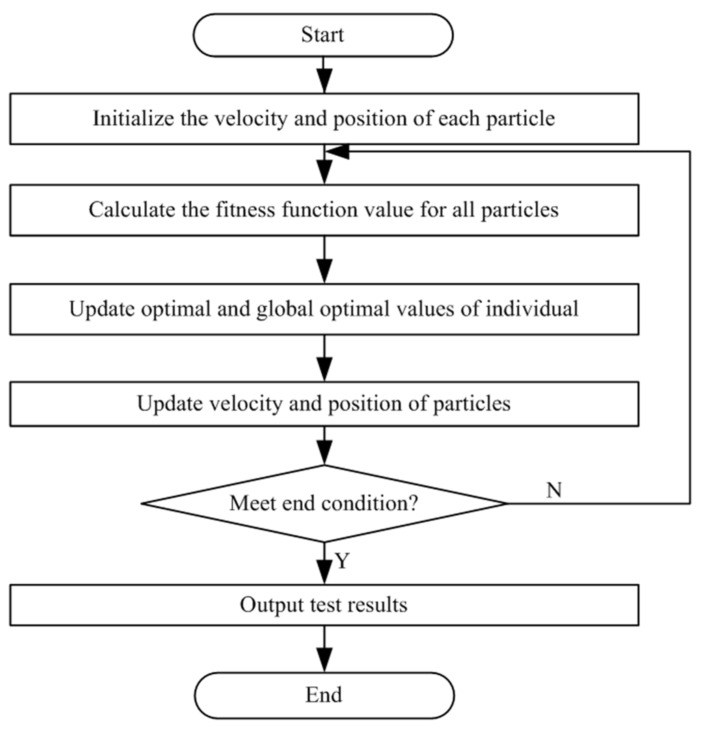
The basic flow of the PSO.

The basic flow of the PSO is shown in Figure 1.

## 3. CLPSO

PSO can easily fall into local extrema, which leads to premature convergence. Thus, a new update strategy is presented to develop a CLPSO algorithm. In the PSO, each particle learns from its own optimal value and the global optimal value. Therefore, in the velocity update formula of CLPSO, the social part of the global optimal solution of particle learning is not used. In addition, in the velocity update formula of the traditional PSO algorithm, each particle learns from all dimensions of its own optimal value, but its own optimal value is not optimal in all dimensions. Therefore, the CLPSO algorithm introduces a comprehensive learning strategy to construct learning samples using the pBest of all particles to promote the information exchange, improve population diversity, and avoid falling into local extrema. The comprehensive learning strategy is to use the individual historical optimal solution of all particles in the population to update the particle position in order to effectively enhance the exploration ability of the PSO and achieve excellent optimization performance in solving multimodal optimization problems. The velocity update of particle and position is described as follows:

(3) $$\{\begin{array}{c} v_{ij}^{t+1}=\omega v_{ij}^{t}+cr_{ij}^{t}\left(pBest_{f_{i}\left(j\right)}^{t}-x_{ij}^{t}\right)\\ v_{ij}^{t+1}=min\left(v_{ijmax}^{},\max\left(v_{ijmin}^{t},v_{ij}^{t+1}\right)\right)\\ x_{ij}^{t+1}=x_{ij}^{t}+v_{ij}^{t+1}\\ x_{ij}^{t+1}=min\left(x_{ijmax}^{},\max\left(x_{ijmin}^{},x_{ij}^{t+1}\right)\right) \end{array}$$

where i=1,2,3,⋯, P and j=1,2,3,⋯, *D*. P is the size of the population and D is the search space dimension. $x_{i}^{t}=[x_{i1}^{t},x_{i2}^{t},\cdots ,x_{ij}^{t},\cdots ,x_{iD}^{t}]$ is the particle position,$v_{i}^{t}=[v_{i1}^{t},v_{i2}^{t},\cdots ,v_{ij}^{t},\cdots ,v_{iD}^{t}]$ is the velocity of particle i, $\left[x_{ijmin}^{},x_{ijmax}^{}\right]$ is the search range of particle i, $\left[v_{ijmin}^{},v_{ijmax}^{}\right]$ is the velocity range, ω is the inertia weight, c is the learning factor, $r_{ij}^{t}$ is a randomly distributed number on (0, 1), $f_{i}\left(j\right)$ refers to other particles that particle i needs to learn in the D-dimension, and $pbest_{f_{i}\left(j\right)}^{t}$ can be the optimal position of any particle.

The determination method of $f_{i}\left(j\right)$ is described as follows: For each particle dimension, a random probability is produced. If the random probability is greater than the learning probability $P_{c_{i}}$, then this particle dimension learns from the corresponding dimension of its own individual optimal value. On the other hand, two particles are randomly selected to learn the better optimal value. To ensure the population’s polymorphism, the CLPSO also sets an update interval number m; that is, when the individual optimal value of particle i has not been updated for m iterations, it is regenerated.

## 4. MSACLPSO

PSO has simplicity, practicality, and fixed parameters, but it has the disadvantage of easily falling into local optima, as well as weak local search ability. The CLPSO has slow velocity in the later search, low convergence speed, and unstable performance. To solve these problems, a multi-strategy adaptive CLPSO (MSACLPSO) algorithm is proposed by introducing a comprehensive learning strategy, multi-population parallel strategy, velocity update strategy, and parameter adaptive strategy. In MSACLPSO, a comprehensive learning strategy is introduced to construct learning samples using the pBest of all particles to promote information exchange, improve population diversity, and avoid falling into local extrema. To overcome the lack of local search ability in the later stage, the global optimal value of the population is used for the velocity update, and a new update strategy is proposed to enhance the local search ability. The multi-population parallel strategy is employed to divide the population into N subpopulations, and then iterative evolution is carried out appropriately to achieve particle exchange and mutation, enhance the population diversity, accelerate the convergence, and ensure information sharing between the particles. The linearly changing strategy of the learning factors is employed to realize the iterative evolution in different stages and the adaptive adjustment strategy of learning factors, which can enhance the global search ability and improve the local search ability. The S-shaped decreasing function is adopted to realize the adaptive adjustment of inertia weight to ensure that the population has high speed in the initial stage, reduce the search speed in the middle stage—so that the particles will more easily converge to the global optimum—and maintain a certain speed for the final convergence in the later stage.

### 4.1. Multi-Population Parallel Strategy

The idea of multi-population parallel is based on the natural phenomenon of the evolution of the same species in different regions. It divides the population into multiple subpopulations, and then each subpopulation searches for the optimal value in parallel to improve the search ability. The indirect exchange of the optimal value and dynamic recombination of the population can enhance the population diversity and accelerate the convergence. A multi-population parallel strategy is proposed here. The main ideas of the multi-population parallel strategy are described as follows: The population is divided into *N* subpopulations in the process of evolution. For each subpopulation, the particle carries out iterative evolution, and the particle exchange and particle mutation under appropriate conditions are executed according to certain rules, so as to ensure information sharing between the particles of the population through the exchange of particles between subpopulations. Therefore, to enhance the local search ability of the CLSPO algorithm in the later stage, a new update strategy is presented after the g0 generation is completed. That is, the global optimal value gBest of the population is added to the velocity update, as shown in Equation (4):

(4) $$\{\begin{array}{c} v_{ij}^{t+1}=\omega v_{ij}^{t}+c_{1}r_{1ij}^{t}\left(pBest_{f_{i}\left(j\right)}^{t}-x_{ij}^{t}\right)+c_{2}r_{2ij}^{t}\left(gBest_{f_{i}\left(j\right)}^{t}-x_{ij}^{t}\right)\\ v_{ij}^{t+1}=\min\left(v_{ijmax}^{},\max\left(v_{ijmin}^{t},v_{ij}^{t+1}\right)\right)\\ x_{ij}^{t+1}=x_{ij}^{t}+v_{ij}^{t+1}\\ x_{ij}^{t+1}=\min\left(x_{ijmax}^{},\max\left(x_{ijmin}^{},x_{ij}^{t+1}\right)\right) \end{array}$$

where $c_{1}$ and $c_{2}$ are learning factors, $pBest_{f_{i}\left(j\right)}^{t}$ is the optimal value of the particle in each subpopulation $\left\{\;pBest_{1}^{t}\;,pBest_{2}^{t},\cdots ,pBest_{p}^{t},\cdots ,pBest_{P}^{t}\right\}$, $gBest_{f_{i}\left(j\right)}^{t}$ is the optimal value of each subpopulation $\left\{gBest_{1}^{t}\;,gpBest_{2}^{t},\cdots ,gBest_{p}^{t},\cdots ,gBest_{P}^{t}\right\}$, $r_{1ij}^{t}$ and $r_{2ij}^{t}$ are randomly distributed numbers on (0, 1).

### 4.2. Adaptive Learning Factor Strategy

In PSO, the values of $c_{1}$ and $c_{2}$ are set in advance according to experiences, reducing the self-learning ability. Therefore, the linearly changing strategy of the learning factors is developed for $c_{1}$ and $c_{2}$. In the early evolution stage, the self-cognition item is reduced and the social cognition item is increased to improve the global search ability. In the later evolution stage, the local search ability is guaranteed by encouraging particles to converge towards the global optimum. Therefore, the adaptive learning factor strategy is described as follows:

(5) $$c_{1}=c_{min}+\left(c_{max}-c_{min}\right)(T-t)/T$$

(6) $$c_{2}=c_{min}+\left(c_{max}-c_{min}\right)t/T$$

where $c_{max}\;$ and $c_{min}$ are the maximum value and minimum value, respectively.

### 4.3. Adaptive Inertia Weight Strategy

In PSO, when the particles in the population tend to be the same, the last two terms in the particle velocity update formula—namely, the social cognition part, and the individual’s own cognition part—will gradually tend towards 0. If the inertia weight ω is less than 1, the particle speed will gradually decrease, or even stop moving, which result in premature convergence. When the optimal fitness of the population has not changed (i.e., has stagnated) for a long time, the inertia weight ω should be adjusted adaptively according to the degree of premature convergence. If the same adaptive operation is adopted for the population, when the population has converged to the global optimum, the probability of destroyed excellent particles will increase with the increase in their inertia weight, which will degrade the performance of the PSO algorithm. To better balance the search ability, an S-shaped decreasing function is adopted to ensure that the population has high speed in the initial stage, and the search speed decreases in the middle stage, so that the particles can easily converge to the global optimum value and, finally, converge at a certain speed in the later stage. The S-shaped decreasing function for the inertia weight ω is described as follows:

(7) $$\omega =(\omega_{max}-\omega_{min})/\left(1+exp\left(2*a*t/T-a\right)\right)+\omega_{min}$$

where $\omega_{max}$ and $\omega_{min}$ are the maximum and minimum values, respectively—$\omega_{max}=0.9$ and $\omega_{min}=0.2$—and a is the control coefficient to adjust the speed change, where *a* = 13.

### 4.4. Model of MSACLPSO

**Figure 2:**
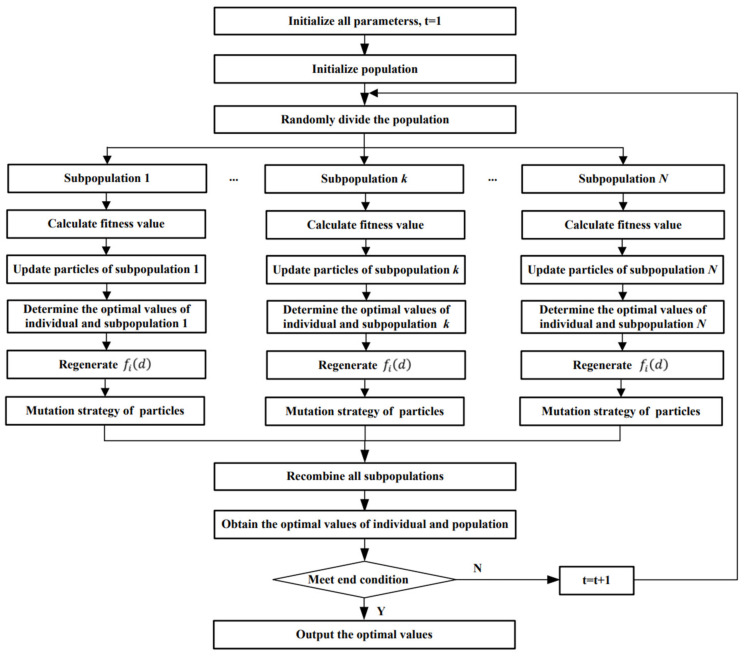
The flow of MSACLPSO.

The flow of MSACLPSO is shown in Figure 2.

The steps of MSACLPSO are described as follows:

**Step 1:** Divide the population into N subpopulations, and initialize all parameters.

**Step 2:** Execute the CLPSO algorithm for each subpopulation. The objective function is used to find out the individual optimal value of the particle, the optimal value of the subpopulation, and the global optimal value of the population. To ensure the high global search ability in the early stage, *T*0 is set for the early stage, and each subpopulation updates all particle states according to Equation (3). To enhance the local search ability of CLSPO in the later stage, after the *T*0 iteration is completed, each subpopulation updates all particle states according to Equation (4).

**Step 3:** If the optimal value of one subpopulation does not update for successive R1 iterations, the population may fall into local optimization. To avoid falling into the local optimum for the subpopulation, the mutation strategy is used here. Each dimension of each particle in the subpopulation is mutated with the probability $P_{m}$. The mutation mode is described as follows:

(8) $$x_{id}^{t}=x_{id}^{t}+randn\left(x_{idmax}^{}-x_{idmin}^{}\right)(T-t)/T$$

where randn is the random number on (−1, 1).

**Step 4:** After *T*0 iterations are executed, to enhance population diversity, the particles are randomly exchanged between populations every interval R iteration to recombine subpopulations. The recombination of subpopulations is described as follows: All subpopulations randomly select 50% of the particles, which are randomly exchanged with the particles of other populations. Then, according to the fitness values of all particles in all subpopulations, 1/*N* particles with the best fitness values in each subpopulation are selected to construct a new population. It is worth noting that the exchanged particle can be any particle in any other population.

**Step 5:** Determine whether the end conditions are met. If they are met, the optimal result is output; otherwise, return to Step 2.

## 5. Experiment Simulation and Analysis

### 5.1. Test Functions

To verify the performance of MSACLPSO, 10 famous benchmark functions were selected. The detailed description is shown in Table 1.

### 5.2. Experimental Environment and Parameter Setting

The experimental environment mainly included Core I5-4200H, Win10, RAM-16GB, and MATLAB R2018b. The optimization performance of MSACLPSO was compared with other state-of-the-art algorithms, including the basic version of PSO (PSO) [46], self-organizing hierarchical PSO (HPSO) [47], fully-informed PSO (FIPS) [48], unified PSO (UPSO) [49], CLPSO [30], and static heterogeneous particle swarm optimization (sHPSO) [50]. In MSACLPSO, the population is divided into two subpopulations, and four main parameters are adjusted to balance exploration and exploitation. These parameters include population size, acceleration coefficients, iteration number, and dimensions. In our experiment, a large number of alternative values were tested, and some classical values were selected from other literature, and then these parameter values were experimentally modified until the most reasonable parameter values were selected. These selected parameter values attained the optimal solution, so that they could accurately and efficiently verify the effectiveness of MSACLPSO in solving optimization problems. Some parameters that were tuned included the population size *N_P_* = 40, the number of subpopulations *N* = 2, $c_{\_min}$ = 0.5 and $c_{\_max}$ = 2.5, the dimension *D* = 30, run times *T* = 30, the maximum number of iterations G = 200, and function evaluations FEs = 300,000. The specific settings are shown in Table 2.

### 5.3. Experimental Results and Analysis

The population was divided into two subpopulations, and different numbers of individuals were set. The error mean (mean) value and standard deviation (Std) value were applied to evaluate the optimization performance of MSACLPSO. The obtained experimental results with the different numbers of individuals for 30-dimensional problems are shown in Table 3. The best results are the bold.

As can be seen from Table 3, the subpopulation size $P_{1}$ = 10 and $P_{2}$ = 30 obtained the best optimization performance for the 10 test benchmark functions compared with other subpopulation sizes. However, for the functions $F_{5}$, $F_{6}$, and $F_{9}$, MSACLPSO did not obtain satisfactory optimization performance. Therefore, the subpopulation size $P_{1}$ = 10 and $P_{2}$ = 30 was selected for performance evaluation of MSACLPSO.

MSACLPSO was compared with some variants of PSO algorithms. The optimization performance was obtained according to the mean and Std of the 20 obtained results. The obtained experimental results using different algorithms for test functions with 30 dimensions are shown in Table 4. The obtained best results are highlighted in **bold**.

As shown in Table 4, all algorithms performed equally on test function $F_{1}$, and PSO obtained the best solution on test function $F_{4}$. FIPS obtained the best solution on test function $F_{5}$. MSACLPSO performed well on test functions $F_{1}$~$F_{5}$. For multimodal functions, MSACLPSO performed well on all functions, and obtained the best performance on test functions $F_{7}$, $F_{8}$, and $F_{10}$, and the second-best performance on test functions $F_{6}$ and $F_{9}$. On the other hand, CLPSO and HCLPSO obtained the best solution on test function $F_{6}$, and OLPSO and HCLPSO obtained the best solution on test function $F_{9}$. Overall, MSACLPSO obtained the best performance on 6 out of 10 test functions. Therefore, MSACLPSO performs well, and obtains the best optimization performance for multimodal problems. In our experiment, MSACLPSO used several strategies of comprehensive learning, multi-population parallel, and parameter adaptation. Although the strategies of comprehensive learning and parameter adaptation need more running time, the multi-population parallel strategy can reduce the running time. Therefore, the time complexity of MSACLPSO is similar to that of the other compared algorithms.

To test the statistical difference between MSACLPSO and the other variants of PSO algorithms, the non-parametric Wilcoxon signed-rank test was used to compare the results of MSACLPSO and the results of the other variants of PSO. The obtained results of MSACLPSO against other algorithms are shown in Table 5.

As shown in Table 5, MSACLPSO performs better than the other variants of PSO algorithms through the number of (+/=/−) in the last row of the Wilcoxon signed-rank test results under α = 0.05.

To sum up, it can be seen that the optimized values of parameters for MSACLPSO are ω = 0.43, c = 2.1, $c_{1}$ = 1.8, $c_{2}$ = 2.1, and $P_{c_{i}}$ = 0.5 for solving these complex optimization problems.

## 6. Case Analysis

Renewable energy has always been the focus of dealing with the key issues of traditional energy consumption, which uses nonrenewable energy. Solar energy is an up-and-coming resource, in which PV plays a vital role. However, the PV device is usually placed in an exposed environment, which leads to its degradation. This seriously affects the efficiency of PV. Therefore, MSACLPSO was employed to effectively and accurately optimize the PV parameters to establish an optimized PV model. The values of parameters for MSACLPSO were the same as given in Section 5.3.

### 6.1. Modeling for PV

A lot of PV models have been designed, and were applied to illustrate the I–V characteristics. The SDM and DDM are the most widely used [51]. The PV model is described in Table 6.

It is crucial to search for the optimal parameter values in order to minimize the error of the PV models. The error functions are described as follows: For the SDM,

(9) $$\left\{\begin{array}{l} f(V_{L},I_{L},x)=I_{ph}-I_{sd}\left[exp\left(\frac{q(V_{L}+I_{L}R_{s})}{nkT}\right)-1\right]-\frac{V_{L}+I_{L}R_{s}}{R_{sh}}-I_{L}\\ x=\left\{I_{ph},I_{sd},R_{s},R_{sh},n\right\} \end{array}\right.$$

For the DDM,

(10) $$\left\{\begin{array}{l} f(V_{L},I_{L},x)=I_{ph}-I_{sd1}\left[exp\left(\frac{q(V_{L}+I_{L}R_{s})}{n_{1}kT}\right)-1\right]-I_{sd2}\left[exp\left(\frac{q(V_{L}+I_{L}R_{s})}{n_{2}kT}\right)-1\right]-\frac{V_{L}+I_{L}R_{s}}{R_{sh}}-I_{L}\\ x=\left\{I_{ph},I_{sd1},I_{sd2},R_{s},R_{sh},n_{1},n_{2}\right\} \end{array}\right.$$

To evaluate the PV model, the RMSE is described as follows:

(11) $$RMSE(x)=\sqrt{\frac{1}{N}\sum_{k=1}^{N}f{\left(V_{L},I_{L},x\right)}^{2}}$$

### 6.2. Modeling for PV

To validate the performance of MSACLPSO, the PSO, BLPSO, CLPSO, CPMPSO, IJAYA, GOTLBO, SATLBO, DE/BBO, DBBO, STLBO, WOA, CWOA, LWOA, GWO, EGWO, WDO, DE, JADE, and MPPCEDE [52,53,54,55,56,57,58,59,60,61,62,63,64] algorithms were used for comparison. The parameter values of MSACLPSO were the same as given in Section 5.2. The parameter values of the other compared algorithms were the same as in the literature. The maximum number of iterations was G = 200, and these algorithms were executed for 20 runs. Therefore, the statistical results of the SRE, LRE, MRE, and Std were obtained. The value of RMSE was employed to quantify the solution accuracy, while the Std of the RMSE described the reliability. The statistical results of the experiment with the SDM and DDM are shown in Table 7 and Table 8, respectively. The obtained best results are highlighted in **bold.**

As can be seen from Table 7, CPMPSO, MPPCEDE, and MSACLPSO obtained the SRE, LRE, and MRE values. For the Std of RMSE, MSACLPSO performed well. Therefore, the optimization performance of MSACLPSO was better than that of the compared algorithms for SDM. As can be seen from Table 8, MSACLPSO obtained the best results for the SRE, LRE, MRE, and Std of RMSE. For the Std of RMSE, MSACLPSO obtained the best Std. Therefore, MSACLPSO is the best algorithm for DDM.

To sum up, it can be seen that the performance of MSACLPSO was demonstrated by optimizing the PV model parameters All of the compared results containing the optimized parameters, along with the SRE, LRE, MRE, and Std values, show that MSACLPSO can obtain the optimal parameters. This provides a more effective parameter combination for the complex engineering problems of photovoltaics, so as to improve the energy conversion efficiency.

## 7. Conclusions

In this paper, a multi-strategy adaptive CLPSO with comprehensive learning, multi-population parallel, and parameter adaptation is proposed. A multi-population parallel strategy was designed to improve population diversity and accelerate convergence. Then, a new velocity update strategy was designed for the velocity update, and a new adaptive adjustment strategy of learning factors was developed. Additionally, a parameter optimization method for photovoltaics was designed to prove the actual application ability. Ten benchmark functions were used to prove the effectiveness of MSACLPSO in comparison with different variants of PSO. On 6 out of 10 functions, MSACLPSO obtained the best performance. MSACLPSO performed well and obtained the best optimization performance for multimodal problems. In addition, the actual SDM and DDM were selected for parameter optimization. The experimental results show that the actual application ability of the MSACLPSO was confirmed in comparison with the other algorithms. MSACLPSO is an alternative optimization technique for solving complex problems and actual engineering problems.

However, MSACLPSO is still insufficient in solving large-scale parameter optimization problems, such as time complexity and easy stagnation, among others. In the future, these applications should be considered [65,66,67,68,69,70,71,72]. The algorithm should be deeply studied, and the parameter adaptability of MSACLPSO in different stages and scales should also be further explored in future works.

## Figures and Tables

**Table 1:** The detailed description.

Function Name	Function Expression	S	$F_{min}$	$f_{-}bias$
Sphere	$F_{1}=\sum_{i=1}^{D}x_{i}$	${\left[-100,\;100\right]}^{D}$	0	−450
Schwefel 1.2	$F_{2}=\sum_{i=1}^{D}{\left(\sum_{j=1}^{i}x_{j}\right)}^{2}$	${\left[-100,\;100\right]}^{D}$	0	−450
High Conditioned Elliptic	$F_{3}=\sum_{i=1}^{D}{\left(10^{6}\right)}^{\frac{i-1}{D-1}}x_{i}^{2}$	${\left[-100,\;100\right]}^{D}$	0	−450
Schwefel 1.2 with Noise	$F_{4}=\left(\sum_{i=1}^{D}{\left(\sum_{j=1}^{i}x_{j}\right)}^{2}\right)*\left(1+0.4|N(0,1\right)$	${\left[-100,\;100\right]}^{D}$	0	−450
Schwefel 2.6	$F_{5}=max\left\{\left|x_{1}+2x_{2}-7\right|,\left|2x_{1}+x_{2}-5\right|\right\}$	${\left[-100,\;100\right]}^{D}$	0	−310
Rosenbrock	$F_{6}=\sum_{i=1}^{D-1}(100{\left(x_{i}^{2}-x_{i+1}\right)}^{2}+\left(x_{i}-1{)}^{2}\right)$	${\left[-100,\;100\right]}^{D}$	0	390
Griewank	$F_{7}=\sum_{i=1}^{D-1}\frac{x_{i}^{2}}{4000}-\prod_{i=1}^{D}\cos\left(\frac{x_{i}}{\sqrt{i}}\right)+1$	${\left[-100,\;100\right]}^{D}$	0	390
Ackley	$F_{8}=-20exp\left(-0.2\sqrt{\frac{1}{D}\sum_{i=1}^{D}x_{i}^{2}}\right)-exp\left(\frac{1}{D}\sum_{i=1}^{D}\cos(2\pi x_{i})\right)+20$	${\left[-32,\;32\right]}^{D}$	0	−140
Rastrigin	$F_{9}=\sum_{i=1}^{D}\left(x_{i}^{2}-10\cos\left(2\pi x_{i}\right)+10\right)$	${\left[-5,\;5\right]}^{D}$	0	−330
Expanded Schaffer	$F_{10}=0.5+\frac{\left(sin^{2}\left(\sqrt{x^{2}+y^{2}}\right)-0.5\right)}{{\left(1+0.001\left(x^{2}+y^{2}\right)\right)}^{2}}$	${\left[-100,\;100\right]}^{D}$	0	−300

**Table 2:** The parameter settings.

Algorithms	ω	$c_{}$	$c_{1}$	$c_{2}$	$P_{c_{i}}$	$N_{P}$	FES
PSO	0.9~0.4	—	2.0	2.0	—	60	300,000
HPSO	—	—	2.5~0.5	0.5~2.5	—	40	300,000
FIPS	—	2	—	—	—	40	300,000
UPSO	—	1.49445	—	—	—	40	300,000
OLPSO	0.9~0.4	2	—	—	—	40	300,000
CLPSO	0.9~0.4	1.49445	—	—	0.5	40	300,000
sHPSO	0.72	—	2.5~0.5	0.5~2.5	—	40	300,000
MSACLPSO	0.95~0.3	3.0~1.5	2.5~0.5	0.5~2.5	0.5	40	300,000

**Table 3:** The different numbers of individuals ($P_{1}$ and $P_{2}$) in two subpopulations for MSACLPSO.

Functions	Indices	10 + 30	15 + 25	20 + 20	25 + 15	30 + 10	40 + 0
$F_{1}$	Mean	**0.0000**	**0.0000**	**0.0000**	**0.0000**	**0.0000**	**0.0000**
	Std	**0.0000**	**0.0000**	**0.0000**	**0.0000**	**0.0000**	**0.0000**
$F_{2}$	Mean	**1.9862 × 10^−8^**	1.6547 × 10^−6^	2.2056 × 10^−4^	1.6547 × 10^−2^	3.3274 × 10^−1^	3.7543 × 10^−1^
	Std	**3.5974 × 10^−8^**	1.7430 × 10^−6^	3.3469 × 10^−4^	1.3275 × 10^−2^	4.5401 × 10^−1^	4.7341 × 10^−1^
$F_{3}$	Mean	**5.5673 × 10^−5^**	6.1432 × 10^5^	8.4102 × 10^5^	1.3610 × 10^6^	2.1977 × 10^6^	2.3560 × 10^6^
	Std	**1.7703 × 10^−5^**	2.4205 × 10^5^	3.2359 × 10^5^	6.6034 × 10^5^	9.2605 × 10^5^	6.6496 × 10^5^
$F_{4}$	Mean	**4.2485 × 10^2^**	5.0328 × 10^2^	6.0462 × 10^2^	8.1743 × 10^2^	1.4058 × 10^3^	1.4135 × 10^3^
	Std	**2.4452 × 10^2^**	2.8874 × 10^2^	4.4718 × 10^2^	4.0569 × 10^2^	7.1673 × 10^2^	6.4532 × 10^2^
$F_{5}$	Mean	2.7830 × 10^3^	**2.5065 × 10^3^**	2.8913 × 10^3^	3.1673 × 10^3^	3.2137 × 10^3^3	3.5478 × 10^3^
	Std	5.5702 × 10^2^	4.1407 × 10^2^	**4.0531 × 10^2^**	5.9613 × 10^2^	4.2715 × 10^2^	5.5379 × 10^2^
$F_{6}$	Mean	3.1637	2.2637	**2.1975**	7.1762 × 10^−1^	2.9757 × 10^−1^	3.3405 × 10^−1^
	Std	**3.3643**	4.0623	3.4537	9.2546 × 10^−1^	5.2504 × 10^−1^	6.6492 × 10^−1^
$F_{7}$	Mean	**0.0000**	**0.0000**	**0.0000**	**0.0000**	**0.0000**	**0.0000**
	Std	**0.0000**	**0.0000**	**0.0000**	**0.0000**	**0.0000**	**0.0000**
$F_{8}$	Mean	**1.9745 × 10^1^**	1.9805 × 10^1^	1.9832 × 10^1^	1.9867 × 10^1^	1.9835 × 10^1^	2.0645 × 10^1^
	Std	8.3746 × 10^−2^	7.1485 × 10^−2^	5.8043 × 10^−2^	**5.7903 × 10^−2^**	8.8562 × 10^−2^	7.8530 × 10^−2^
$F_{9}$	Mean	1.0673	1.0245 × 10^−1^	**0.0000**	**0.0000**	**0.0000**	1.2473
	Std	1.0305	3.8672 × 10^−1^	**0.0000**	**0.0000**	**0.0000**	1.2865
$F_{10}$	Mean	**1.0782 × 10^1^**	1.0954 × 10^1^	1.1065 × 10^1^	1.1438 × 10^1^	1.1714 × 10^1^	1.1904 × 10^1^
	Std	**4.0645 × 10^−1^**	5.4680 × 10^−1^	4.3591 × 10^−1^	5.4613 × 10^−1^	4.1527 × 10^−1^	4.3681 × 10^−1^

**Table 4:** The obtained experimental results using different algorithms.

Functions	Indices	PSO	HPSO	FIPS	OLPSO	UPSO	sHPSO	CLPSO	HCLPSO	MSACLPSO
$F_{1}$	Mean	**0.00**	**0.00**	**0.00**	**0.00**	**0.00**	**0.00**	**0.00**	**0.00**	**0.00**
	Std	**0.00**	**0.00**	**0.00**	**0.00**	**0.00**	**0.00**	**0.00**	**0.00**	**0.00**
$F_{2}$	Mean	3.70 × 10^−1^	3.79 × 10^−6^	7.79 × 10^1^	1.38 × 10^1^	2.65E-07	1.44 × 10^−2^	1.14 × 10^3^	1.70 × 10^−6^	**1.99 × 10^−8^**
	Std	3.20 × 10^−1^	2.82 × 10^−6^	2.71 × 10^1^	8.33	2.42E-07	7.10 × 10^−2^	2.53 × 10^2^	1.71 × 10^−6^	**3.60 × 10^−8^**
$F_{3}$	Mean	6.53 × 10^6^	7.72 × 10^5^	2.45 × 10^7^	1.60 × 10^7^	1.54 × 10^6^	8.75 × 10^5^	1.22 × 10^7^	6.42 × 10^5^	**5.57 × 10^5^**
	Std	4.17 × 10^6^	2.96 × 10^5^	6.29 × 10^6^	7.04 × 10^6^	4.75 × 10^5^	5.34 × 10^5^	3.34 × 10^6^	2.61 × 10^5^	**1.77 × 10^5^**
$F_{4}$	Mean	**3.81 × 10^2^**	2.48 × 10^4^	1.15 × 10^3^	2.18 × 10^3^	7.28 × 10^3^	2.02 × 10^4^	8.77 × 10^3^	5.22 × 10^2^	4.25 × 10^2^
	Std	3.31 × 10^2^	5.71 × 10^3^	3.73 × 10^2^	1.09 × 10^3^	2.79 × 10^3^	9.94 × 10^3^	1.85 × 10^3^	3.09 × 10^2^	**2.45 × 10^2^**
$F_{5}$	Mean	3.85 × 10^3^	9.20 × 10^3^	**2.22 × 10^3^**	3.30 × 10^3^	6.32 × 10^3^	6.94 × 10^3^	4.47 × 10^3^	2.97 × 10^3^	2.78 × 10^3^
	Std	8.00 × 10^2^	1.81 × 10^3^	5.14 × 10^2^	**3.75 × 10^2^**	1.63 × 10^3^	1.43 × 10^3^	4.26 × 10^2^	4.55 × 10^2^	5.57 × 10^2^
$F_{6}$	Mean	7.02 × 10^1^	5.04 × 10^1^	3.77 × 10^1^	2.07 × 10^1^	6.82 × 10^1^	1.15 × 10^2^	**2.39**	**2.39**	3.16
	Std	9.51 × 10^1^	5.05 × 10^1^	3.50 × 10^1^	2.50 × 10^1^	9.64 × 10^1^	2.29 × 10^2^	3.84	4.27	**3.36**
$F_{7}$	Mean	7.60 × 10^−1^	1.00 × 10^−2^	3.00 × 10^−2^	1.00 × 10^−2^	2.00 × 10^−2^	4.00 × 10^2^	7.00 × 10^−1^	2.00 × 10^−2^	**0.00**
	Std	1.41	1.00 × 10^−2^	2.00 × 10^−2^	1.00 × 10^−2^	1.00 × 10^−2^	4.00 × 10^2^	1.50 × 10^−1^	2.00 × 10^−2^	**0.00**
$F_{8}$	Mean	2.09 × 10^1^	2.07 × 10^1^	2.09 × 10^1^	2.10 × 10^1^	2.10 × 10^1^	2.02 × 10^1^	2.10 × 10^1^	2.09 × 10^1^	**1.97 × 10^1^**
	Std	7.00 × 10^−2^	1.50 × 10^−1^	6.00 × 10^−2^	8.00 × 10^−2^	**5.00 × 10^−2^**	1.90 × 10^−1^	6.00 × 10^−2^	9.00 × 10^−2^	8.37 × 10^−2^
$F_{9}$	Mean	1.90 × 10^1^	1.07 × 10^1^	5.71 × 10^1^	**0.00**	8.52 × 10^1^	8.25 × 10^1^	1.00 × 10^2^	**0.00**	1.07
	Std	5.37	4.96	1.46 × 10^1^	**0.00**	1.69 × 10^1^	2.44 × 10^1^	1.25 × 10^1^	**0.00**	1.03
$F_{10}$	Mean	1.27 × 10^1^	1.23 × 10^1^	1.31 × 10^1^	1.31 × 10^1^	1.28 × 10^1^	1.31 × 10^1^	1.26 × 10^1^	1.19 × 10^1^	**1.09 × 10^1^**
	Std	4.30 × 10^−1^	3.70 × 10^−1^	2.10 × 10^−1^	**2.00 × 10^−1^**	3.30 × 10^−1^	3.90 × 10^−1^	2.20 × 10^−1^	5.80 × 10^−1^	4.06 × 10^−1^

**Table 5:** The test results under α = 0.05.

Functions	PSO	HPSO	FIPS	OLPSO	UPSO	sHPSO	CLPSO	HCLPSO
$F_{1}$	**=**	**=**	**=**	**=**	**=**	**=**	**=**	**=**
$F_{2}$	**+**	**+**	**+**	**+**	**+**	**+**	**+**	**+**
$F_{3}$	**+**	**+**	**+**	**+**	**+**	**+**	**+**	**+**
$F_{4}$	−	**+**	**+**	**+**	**+**	**+**	**+**	**+**
$F_{5}$	**+**	**+**	−	**+**	**+**	**+**	**+**	**+**
$F_{6}$	**+**	**+**	**+**	**+**	**+**	**+**	−	−
$F_{7}$	**+**	**+**	**+**	**+**	**+**	**+**	**+**	**+**
$F_{8}$	**+**	**+**	**+**	**+**	**+**	**+**	**+**	**+**
$F_{9}$	**+**	**+**	**+**	−	**+**	**+**	**+**	−
$F_{10}$	**+**	**+**	**+**	**+**	**+**	**+**	**+**	**+**
+/=/−	**8/1/1**	**9/1/0**	**8/1/1**	**8/1/1**	**9/1/0**	**9/1/0**	**8/1/1**	**7/1/2**

**Table 6:** The modelling for PV.

PV	$I_{L}$
SDM	$I_{L}=I_{ph}-I_{sd}\left[exp\left(\frac{q(V_{L}+I_{L}R_{s})}{nkT}\right)-1\right]-\frac{V_{L}+I_{L}R_{s}}{R_{sh}}$
DDM	$I_{L}=I_{ph}-I_{sd1}\left[exp\left(\frac{q(V_{L}+I_{L}R_{s})}{n_{1}kT}\right)-1\right]-\;I_{sd2}\left[exp\left(\frac{q(V_{L}+I_{L}R_{s})}{n_{2}kT}\right)-1\right]-\frac{V_{L}+I_{L}R_{s}}{R_{sh}}$

**Table 7:** The obtained results of RMSE for the SDM.

Algorithms	SRE	LRE	MRE	Std	Symbol
PSO	2.44805 × 10^−3^	**9.86022 × 10^−4^**	1.31844 × 10^−3^	5.24500 × 10^−4^	+
BLPSO	1.74592 × 10^−3^	1.03122 × 10^−3^	1.31377 × 10^−3^	1.90400 × 10^−4^	+
CLPSO	1.25274 × 10^−3^	9.92075 × 10^−4^	1.06081 × 10^−3^	7.04200 × 10^−5^	+
CPMPSO	**9.86022 × 10^−4^**	**9.86022 × 10^−4^**	**9.86022 × 10^−4^**	2.17556 × 10^−17^	+
IJAYA	9.86841 × 10^−4^	**9.86022 × 10^−4^**	9.86051 × 10^−4^	1.49300 × 10^−7^	+
GOTLBO	1.39559 × 10^−3^	9.86608 × 10^−4^	1.08300 × 10^−3^	9.70900 × 10^−5^	+
SATLBO	1.00674 × 10^−3^	9.86025 × 10^−4^	9.88799 × 10^−4^	4.81300 × 10^−6^	+
DE/BBO	1.84123 × 10^−3^	**9.86022 × 10^−4^**	1.25173 × 10^−3^	2.08225 × 10^−4^	+
DBBO	2.36083 × 10^−3^	9.86820 × 10^−4^	1.38755 × 10^−3^	2.70008 × 10^−4^	+
STLBO	1.02033 × 10^−3^	**9.86022 × 10^−4^**	9.87207 × 10^−4^	6.25700 × 10^−6^	+
WOA	1.00397 × 10^−2^	1.10759 × 10^−3^	3.25587 × 10^−3^	2.16463 × 10^−3^	+
CWOA	3.28588 × 10^−2^	9.98677 × 10^−4^	5.44921 × 10^−3^	6.33831 × 10^−3^	+
LWOA	1.92042 × 10^−2^	9.99621 × 10^−4^	3.44545 × 10^−3^	3.33774 × 10^−3^	+
GWO	4.43070 × 10^−2^	1.28030 × 10^−3^	1.13440 × 10^−2^	1.48470 × 10^−2^	+
EGWO	5.24900 × 10^−3^	2.11210 × 10^−3^	3.50150 × 10^−3^	1.59880 × 10^−3^	+
WDO	4.42600 × 10^−3^	1.22101 × 10^−3^	2.18020 × 10^−3^	7.63880 × 10^−4^	+
DE	1.81059 × 10^−3^	**9.86022 × 10^−4^**	1.02116 × 10^−3^	1.44688 × 10^−4^	+
JADE	1.41030 × 10^−3^	9.86060 × 10^−4^	1.08330 × 10^−3^	1.09000 × 10^−4^	+
MPPCEDE	**9.86022 × 10^−4^**	**9.86022 × 10^−4^**	**9.86022 × 10^−4^**	**0.00000**	+
MSACLPSO	**9.86022 × 10^−4^**	**9.864574 × 10^−4^**	**9.83758 × 10^−4^**	**7.52967 × 10^−18^**	

**Table 8:** The obtained results of the RMSE for the DDM.

Algorithms	SRE	LRE	MRE	Std	Symbol
PSO	4.34952 × 10^−2^	**9.82485 × 10^−4^**	4.37645 × 10^−3^	1.01270 × 10^−2^	+
BLPSO	1.93654 × 10^−3^	1.08218 × 10^−3^	1.53462 × 10^−3^	2.45890 × 10^−4^	+
CLPSO	1.38835 × 10^−3^	9.94316 × 10^−4^	1.13959 × 10^−3^	9.39950 × 10^−5^	+
CPMPSO	9.86022 × 10^−4^	**9.82485 × 10^−4^**	9.83137 × 10^−4^	1.33980 × 10^−6^	+
IJAYA	9.99410 × 10^−4^	9.82494 × 10^−4^	9.86860 × 10^−4^	3.22120 × 10^−6^	+
GOTLBO	1.53359 × 10^−3^	9.85097 × 10^−4^	1.16335 × 10^−3^	1.51770 × 10^−4^	+
SATLBO	1.23062 × 10^−3^	9.82824 × 10^−4^	1.00544 × 10^−3^	5.02710 × 10^−5^	+
DE/BBO	1.63508 × 10^−3^	9.87990 × 10^−4^	1.19281 × 10^−3^	2.03849 × 10^−4^	+
DBBO	9.84995 × 10^−4^	2.29052 × 10^−3^	1.22395 × 10^−3^	3.08780 × 10^−4^	+
STLBO	1.52433 × 10^−3^	9.82561 × 10^−4^	1.03435 × 10^−3^	1.41980 × 10^−4^	+
WOA	1.15633 × 10^−3^	1.16011 × 10^−2^	3.42961 × 10^−3^	2.23226 × 10^−3^	+
CWOA	8.86567 × 10^−3^	1.13004 × 10^−3^	3.50587 × 10^−3^	2.15341 × 10^−3^	+
LWOA	1.04935 × 10^−3^	1.11900 × 10^−2^	3.12337 × 10^−3^	1.81559 × 10^−3^	+
GWO	4.07970 × 10^−2^	1.02742 × 10^−3^	9.90850 × 10^−4^	1.29040 × 10^−2^	+
EGWO	5.00690 × 10^−3^	1.80620 × 10^−3^	3.06700 × 10^−3^	1.70500 × 10^−3^	+
WDO	4.93450 × 10^−3^	1.68120 × 10^−3^	3.29180 × 10^−3^	8.41370 × 10^−4^	+
DE	2.00941 × 10^−3^	9.82936 × 10^−4^	1.06862 × 10^−3^	2.23325 × 10^−4^	+
JADE	2.23830 × 10^−3^	9.83510 × 10^−4^	1.46570 × 10^−3^	3.81000 × 10^−4^	+
MPPCEDE	**9.82908 × 10^−4^**	**9.82485 × 10^−4^**	**9.82504 × 10^−4^**	**8.02951 × 10^−8^**	+
MSACLPSO	**9.82743 × 10^−4^**	**9.82368 × 10^−4^**	**9.81463 × 10^−4^**	**9.68924 × 10^−9^**	

## Data Availability

Not applicable.

## Nomenclature

PSO	Particle swarm optimization
HPSO	Hierarchical PSO
FIPS	Fully-informed PSO
UPSO	Unified PSO
sHPSO	Static heterogeneous PSO
CLPSO	Comprehensive learning PSO
MSACLPSO	Multi-strategy adaptive CLPSO
ABC	Artificial bee colony
SMA	Slime mold algorithm
HHO	Harris hawk optimization
w	Inertia weight factor
$c_{1}$	Self-cognition factor
$c_{2}$	Social cognition factor
$V_{\max}$	Max velocity
RMSE	Root-mean-square error
SRE	Smallest RMSE
LRE	Largest RMSE
MRE	Mean RMSE
Std	Standard deviation
PV	Photovoltaics
SDM	Single-diode model
DDM	Double-diode model
I–V	Current–voltage
P–V	Power–voltage
$r_{1}$,$r_{2}$	Random numbers
$v_{ij}^{t+1}$	Velocity
P	Size of population
D	Search space dimension
